# Causes of Bacterial Infections and Antibiotic Resistance Profiles in Patients with Chronic Wounds

**DOI:** 10.3390/microorganisms14091978

**Published:** 2026-09-08

**Authors:** Meltem Yılmaz, Onur Ozturk

**Affiliations:** Department of Family Medicine, Faculty of Medicine, Amasya University, Amasya 05100, Türkiye; dr.onurozturk@yahoo.com

**Keywords:** wound infections, diabetic foot, pressure ulcer, Gram-negative bacteria

## Abstract

This retrospective, descriptive study examined the microbial distribution and antibiotic resistance profiles of predominant pathogens in chronic wounds among patients presenting to a regional hospital’s chronic wound outpatient clinic between July and November 2025, with the aim of informing empirical antibiotic therapy and regional antibiotic stewardship programs. Among the 105 patients included (mean age 62.69 ± 17.14 years, 60% male), diabetic foot ulcer was the most common wound type (49.5%). Of the isolates obtained, 70.2% (n = 73) were Gram-negative and 29.8% (n = 31) were Gram-positive microorganisms, with *Escherichia coli* and *Pseudomonas aeruginosa* being the most frequently isolated pathogens (16.3% each), followed by *Staphylococcus aureus* (15.4%). *S. aureus* predominated in diabetic foot ulcers, whereas *P. aeruginosa* predominated in peripheral artery disease, though no statistically significant association was found between wound type and bacterial group (*p* = 0.055). A multidrug-resistant phenotype was detected in 16.3% (17/104) of isolates. No significant differences emerged between Gram-negative and Gram-positive groups regarding HbA1c, WBC, and CRP levels (*p* > 0.05), although WBC and CRP showed a moderate positive correlation (ρ = 0.396, *p* < 0.001). These findings highlight the predominance of Gram-negative microorganisms and considerable antibiotic resistance in chronic wound infections, emphasizing that culture-based diagnosis remains essential since laboratory parameters cannot reliably predict the causative microbial group, and that empirical therapy should be guided by local resistance patterns.

## 1. Introduction

Chronic wound is defined as a condition in which a wound fails to complete its normal healing stages within a period typically considered to be four to six weeks. A chronic wound infection, on the other hand, occurs when microorganisms invade and multiply within the wound bed, overcoming the host’s defenses and triggering a local or systemic response that impedes healing. This condition is conceptually distinct from bacterial contamination and colonization, in which microorganisms are present in the wound area but do not elicit a host response [1]. Chronic wounds are a significant public health problem characterized by prolonged healing times, frequent recurrent infections, increased morbidity, and high care costs, and they place a serious economic burden on the healthcare system, particularly at the primary care level [2,3]. The prevalence of chronic wounds is rising worldwide due to an aging population and an increase in diabetes and vascular diseases, and related infections are on the rise globally [4,5]. While Gram-negative microorganisms are predominant in chronic wounds, *Staphylococcus aureus* is among the most frequently isolated pathogens [6,7,8,9,10]. In series of diabetic foot infections reported from Turkey, the predominance of Gram-negative pathogens and multidrug-resistant strains suggests that these may take priority over broad-spectrum regimens in empirical treatment [8].

Chronic wounds at the family medicine level place a significant burden on patients, their families, and the primary care team due to frequent visits, long follow-up periods, and the need for dressing changes and monitoring [3]. Effective chronic wound management in primary care is considered one of the core responsibilities of the family medicine discipline due to its potential to reduce amputations, hospitalizations, and costs [3,11].

This study aims to identify the local microbiological distribution and antibiotic resistance profiles in chronic wound infections, to contribute to the development of empirical treatment approaches in family medicine practice, and to strengthen regional antibiotic stewardship programs.

## 2. Materials and Methods

This is a retrospective, descriptive study. The study included patients who attended the chronic wound outpatient clinic at Amasya Sabuncuoğlu Şerefeddin Training and Research Hospital between 1 July and 1 November 2025 and from whom deep tissue samples and microbiological wound culture samples were taken. Patients under the age of 18, those with incomplete data in their medical records, and those from whom no deep tissue sample was taken were excluded from the study.

The material analyzed consisted of swabs taken from chronic wounds using the Levine method. The samples for the test were collected by the permanent doctor working at the chronic wound outpatient clinic. Swab samples obtained from wound sites were assessed for leukocyte and microorganism presence by preparing a Gram stain preparation. Samples were simultaneously inoculated onto 5% sheep blood agar, chocolate agar, and eosin methylene blue agar (EMB) medium (RDS med, Istanbul, Türkiye), and were incubated for 48 h at 37 °C. A VITEK 2 Compact^®^ (bioMérieux, Marcy-l’Étoile, France) fully automated identification system was used for the identification of bacterial growth and antibiotic susceptibility. Additionally, in situations where the automated system could not be utilized, the catalase, coagulase, and PYR tests were used for Gram-positive bacteria, and conventional methods such as motility, indole, urea, and oxidase were used for Gram-negative bacteria. Antibiotic susceptibility tests were performed with the Kirby–Bauer disc-diffusion method by spreading bacterial suspension adjusted to 0.5 McFarland over the surface of a Mueller–Hinton agar medium. The presence of ESBL in *E. coli* and *K. pneumoniae* isolates was investigated by the double disc synergy method. Tests were performed and evaluated in accordance with EUCAST recommendations [12]. *E. coli* ATCC 25922 and *S. aureus* ATCC 29213 were used as a reference strain in antimicrobial susceptibility testing. If cultures showed mixed bacterial growth with significant presence of epithelial cells, specimens were evaluated and reported as elements of the normal skin flora.

The sample size calculation for this study was based on *Staphylococcus aureus*, the pathogen most frequently isolated in wound infections according to the literature [4,13]. The primary reason for this was to ensure that the study would have sufficient statistical power for subgroup analyses to be conducted for different microorganisms. The sample size was calculated using the G*Power 3.1 software. In this context, the effect size w = 0.30 (moderate effect), the significance level α = 0.05, the statistical power (1 − β) = 0.80, and Df = 1 were assumed [14]. As a result of the analysis, the required minimum sample size was calculated to be n = 88. All patients from whom wound culture samples were collected at the chronic wound clinic between July and November 2025 were included in the study.

Data were retrospectively retrieved from the hospital information management system (Sisoft Nucleus v.2.05.725) and patient records. The presence of growth in microbiological wound cultures, the types of microorganisms that grew, and the antibiotic susceptibility and resistance profiles of these microorganisms were examined. It also includes demographic information on patients (age, gender), chronic conditions, particularly the presence of type 2 diabetes mellitus (DM), laboratory parameters such as white blood cell count, CRP, and HbA1c, whether patients were hospitalized or treated on an outpatient basis, and whether a follow-up culture was taken.

Statistical analyses were performed using IBM SPSS Statistics for Windows, Version 25.0 (IBM Corp., Armonk, NY, USA). Descriptive statistics were presented as mean ± standard deviation for continuous variables and as frequencies and percentages for categorical variables. The normality of the data distribution was assessed using the Shapiro–Wilk test and histograms. Associations between categorical variables were evaluated using the Pearson chi-square test or Fisher’s exact test, as appropriate. The Shapiro–Wilk test indicated that HbA1c, WBC, and CRP levels were not normally distributed (*p* < 0.05). Therefore, the Mann–Whitney U test was used for comparisons between two independent groups. Spearman’s rank correlation analysis was performed to assess the relationships among HbA1c, WBC, and CRP levels. A *p*-value < 0.05 was considered statistically significant.

## 3. Results

### 3.1. Demographic and Clinical Characteristics

A total of 105 patients from whom wound cultures were obtained were included in the study. The demographic and clinical characteristics of the patients are presented in Table 1. The median age of the patients was 62 years (range: 18–96 years), and 63 (60.0%) were male. Diabetes mellitus was present in 68 patients (64.8%), hypertension in 35 (33.3%), coronary artery disease in 35 (33.3%), chronic kidney disease in 9 (8.6%), and Alzheimer’s disease/dementia in 7 (6.7%). The most common type of wound was the diabetic foot ulcer (52/105, 49.5%); this was followed by wounds associated with peripheral artery disease (30/105, 28.6%) and pressure ulcers (15/105, 14.3%). The vast majority of patients (97/105, 92.4%) were treated on an outpatient basis.

### 3.2. Microbiological Findings of Wound Cultures 

Of the wound cultures, 70 (66.7%) yielded a single microorganism, 17 (16.2%) yielded two different microorganisms, and 18 (17.1%) showed no microbial growth. From the 87 samples with positive culture results, a total of 104 microbial isolates were obtained, including 70 isolates containing a single microorganism and 34 isolates obtained from polymicrobial cultures.

Of the 104 microorganisms isolated, 73 (70.2%) were Gram-negative and 31 (29.8%) were Gram-positive. The most frequently isolated microorganisms were *Escherichia coli* (17/104; 16.3%) and *Pseudomonas aeruginosa* (17/104, 16.3%), followed by *Staphylococcus aureus* (16/104, 15.4%), *Proteus* spp. (13/104, 12.5%), and *Klebsiella* spp. (11/104; 10.6%). In 8 of the 11 cultures in which *Klebsiella* spp. growth was detected (72.7%), the causative organism was identified as *K. pneumoniae*. The complete distribution of microorganisms isolated from wound cultures is presented in Table 2.

### 3.3. Antimicrobial Resistance Among Gram-Negative Isolates

The antimicrobial resistance profiles of the 73 Gram-negative isolates are presented in Table 3. Resistance was observed against various commonly used antimicrobial agents, with the highest numbers of resistant isolates found for ampicillin (37/73; 50.7%), ciprofloxacin (33/73; 45.2%), amoxicillin–clavulanate (26/73; 35.6%), trimethoprim–sulfamethoxazole (21/73; 28.8%), and piperacillin–tazobactam (18/73; 24.7%).

### 3.4. Antimicrobial Resistance Among Gram-Positive Isolates

The antimicrobial resistance profiles of the 31 Gram-positive isolates are presented in Table 4. The highest resistance rates were observed for tetracycline (41.9%; 13/31), benzylpenicillin (35.5%; 11/31), and clindamycin (35.5%; 11/31). Resistance to fusidic acid was detected in six isolates (19.4%), while resistance to levofloxacin and erythromycin was observed in four isolates each (12.9%).

### 3.5. Multidrug Resistance (MDR) and Mechanisms of Resistance

Of the 104 microbial isolates, 17 (16.3%) exhibited a multidrug-resistant (MDR) phenotype. Methicillin-resistant Staphylococcus aureus (MRSA) accounted for 2 of the 16 S. aureus isolates (12.5%). Extended-spectrum β-lactamase (ESBL) production was detected in 6 of 17 *E. coli* isolates (35.3%) and in 3 of the *K. pneumoniae* isolates (37.5%). Carbapenem resistance was detected in 2 of 17 *P. aeruginosa* isolates (11.8%) and in 4 of 6 *Acinetobacter baumannii* isolates (66.7%).

### 3.6. Distribution of Isolates by Wound Type

The distribution of 104 microbial isolates by wound type is shown in Table 5. Overall, the most frequently isolated microorganisms were *E. coli* (17/104; 16.3%) and *P. aeruginosa* (17/104; 16.3%).

When analyzed by wound type, the microorganisms most frequently isolated from samples derived from diabetic foot ulcers were *S. aureus* (12/54; 22.2%) and *E. coli* (11/54; 20.4%). In samples associated with peripheral artery disease, the most frequently isolated microorganisms were *P. aeruginosa* (7/31; 22.6%) and *Klebsiella* spp. (4/31; 12.9%). Among isolates obtained from pressure ulcers, the predominant microorganisms were *E. coli* (5/14; 35.7%) and *P. aeruginosa* (4/14; 28.6%).

### 3.7. Distribution of Gram-Negative and Gram-Positive Isolates by Wound Type

The distribution of Gram-negative and Gram-positive isolates by wound type is presented in Table 6. Of the isolates obtained from samples associated with diabetic foot ulcers, 34 (63.0%) were Gram-negative and 20 (37.0%) were Gram-positive. Of the isolates obtained from wounds associated with peripheral artery disease, 24 (77.4%) were Gram-negative and 7 (22.6%) were Gram-positive. Thirteen (92.9%) of the isolates obtained from pressure ulcers were Gram-negative microorganisms, while one (7.1%) was Gram-positive. Among the isolates obtained from wounds associated with peripheral venous insufficiency, one (33.3%) was Gram-negative and two (66.7%) were Gram-positive. Among the isolates classified as “other wound types,” one (50.0%) was Gram-negative and one (50.0%) was Gram-positive.

There was no statistically significant association between wound type and Gram stain category (*p* = 0.055).

### 3.8. Comparison of Laboratory Parameters Between Gram-Negative and Gram-Positive Groups

HbA1c, WBC, and CRP levels were compared between patients with Gram-negative and Gram-positive bacterial growth using the Mann–Whitney U test. No statistically significant differences were observed between the two groups in terms of HbA1c (U = 1005.500; *p* = 0.737), WBC (U = 1126.500; *p* = 0.972), or CRP (U = 1079.500; *p* = 0.712).

### 3.9. Correlations Between HbA1c, WBC, and CRP

The relationships between HbA1c, WBC, and CRP were evaluated. Although a weak positive correlation was found between HbA1c and WBC (ρ = 0.140; *p* = 0.131) and between HbA1c and CRP (ρ = 0.161; *p* = 0.081), these relationships were not statistically significant. In contrast, a moderate, positive, and statistically significant relationship was found between WBC and CRP (ρ = 0.396; *p* < 0.001).

## 4. Discussion

The global prevalence of chronic wounds, developing due to various underlying factors, is increasing significantly and poses a major health burden. Microbial colonization is considered one of the most significant factors hindering chronic wound healing [15]. The microbial distribution and antibiotic resistance profiles in chronic wound infections vary by region and institution, which complicates the implementation of empirical treatment. The current study examines the microbiological distribution and antibiotic resistance profiles observed in patients with various types of wounds followed at a chronic wound clinic within a tertiary care hospital, thereby evaluating the role of a culture-based approach in the management of chronic wound infections.

In the current study, a higher proportion of male patients was observed, and the most common type of chronic wound was diabetic foot ulcers (Table 1). The finding that the most common type of chronic wound in previous studies was diabetic foot ulcers, followed by pressure ulcers, venous ulcers, and peripheral artery disease, is consistent with the results of the current study [4,16,17,18]. This finding is consistent with previous studies reporting that chronic wounds are more common, particularly among older adults and males, that their treatment is more challenging, and that they are associated with high treatment costs [19,20].

Microbial growth was detected in 82.9% of the cultures obtained from patients (Table 1); this rate was higher than the 60–75% rates of microbial growth reported in studies on chronic wound cultures [6,21]. Polymicrobial growth was observed in 16.2% of patients (Table 1); this is consistent with the literature, which indicates that polymicrobial infections occur with non-negligible frequency in chronic wounds and even reach rates of up to 25% in some case series [4,13,22]. The higher prevalence of monomicrobial infections is consistent with the literature. In China, the rate of monomicrobial infections was reported to be 86.6% in 815 patients with chronic wounds [4]. However, there are also studies in the literature—such as a Cameroonian cohort with a rate of 44.3% [23] and a Brazilian multicenter study with a rate of 40.6% [24]—that indicate polymicrobial infections are more prevalent than in the current study. These differences in prevalence may be related to the characteristics of the selected samples, as well as geographic and clinical differences.

In the current study, the marked predominance of Gram-negative microorganisms (70.2%, Table 2) was notable; this finding is consistent with studies showing that Gram-negative microorganisms can predominate in chronic wounds and severely infected diabetic foot ulcers [25,26]. The most frequently isolated microorganisms were found to be *E. coli* and *P. aeruginosa* (16.3% each, Table 2). However, the fact that *S. aureus* (22.2%, Table 5) was the most frequently isolated pathogen in diabetic foot ulcers indicates that the microbiological distribution may vary depending on the etiology of the wound. Although global meta-analysis data identify *S. aureus* as the most commonly isolated pathogen in diabetic foot infections, they also reveal that Pseudomonas spp. and *E. coli* are among the major pathogens due to their high prevalence [27]. Studies conducted in India and other regions of Asia emphasize that Gram-negative pathogens, particularly Pseudomonas species, are predominant, and that the microbial profile may vary depending on geographic region, history of antibiotic use, healthcare dynamics, and the stage of the wound [27,28,29]. In multicenter studies with large sample sizes, *S. aureus* alone accounts for one-third of all pathogens, while Gram-negative bacteria account for more than half of all pathogens [4,21].

The distribution of pathogens may vary depending on the wound site from which the culture was obtained, the underlying disease, and the duration of the wound [30]. In the current study, *P. aeruginosa* (22.6%, Table 5) was the most frequently isolated pathogen in peripheral artery disease. Similarly, a study conducted in Kuwait reported that Gram-negative bacteria, particularly *P. aeruginosa*, were more common in ulcers accompanied by ischemia, whereas Gram-positive bacteria were more prevalent in more superficial, non-ischemic wounds [22]. The predominance of Gram-negative bacteria in pressure ulcers (92.9%) is supported by findings in the literature indicating that the distribution of causative agents shifts toward Gram-negative bacteria as wound depth and stage increase [25,31]. The absence of a statistically significant association between wound type and bacterial group in the current study (*p* = 0.055, Table 6) may be related to the lack of a true association, a limited sample size, low numbers in some subgroups, and heterogeneous wound biology.

The particularly high level of antimicrobial resistance in Gram-negative bacteria is one of the key factors complicating the treatment of chronic wound infections. The high rates of resistance to ampicillin, certain cephalosporins, and fluoroquinolones in Gram-negative bacteria are consistent with the resistance rates reported in chronic wound infections [4]. In contrast, the observation of low resistance to amikacin is consistent with the literature [4,32]. Among Gram-negative bacteria, high levels of resistance to amoxicillin–clavulanate were observed, particularly in *Citrobacter* spp. (66.7%, Table 3), *Proteus* spp. (61.5%, Table 3), and *E. coli* (58.8%, Table 3). A review conducted in Africa reported resistance to amoxicillin–clavulanate of 80% in *E. coli* and *Klebsiella* spp. [33]. The fact that carbapenem resistance has not completely disappeared in *P. aeruginosa* and *A. baumannii* is consistent with the current classification of these pathogens as critically important resistant species in chronic wounds [33,34]. Although resistance to benzylpenicillin, clindamycin, and tetracycline was prominent among Gram-positive bacteria, the lower rates of resistance to glycopeptides and newer agents are consistent with previous studies. In large cohorts, resistance to penicillin, erythromycin, and clindamycin was high among *S. aureus* isolates, while susceptibility to vancomycin was preserved [4,25]. These findings underscore the need for empirical treatment to be based on local antibiotic susceptibility data and reinforce the importance of a culture-based approach.

The MDR rate among the isolated microorganisms was found to be 16.3%, which is similar to the MDR rate (16.8%) reported in the study by Chen et al. [35]. A more extensive meta-analysis reported a much higher prevalence of MDR, particularly in diabetic foot ulcers; this indicates that the frequency of MDR varies significantly depending on wound type, patient profile, prior antibiotic exposure, and level of care [36]. It is well established in the literature that Gram-negative bacteria are more likely to develop multidrug resistance (MDR) than Gram-positive bacteria [36,37]; in the current study, this finding is supported by the high rates of ESBL and carbapenem resistance. This finding suggests that the use of empirical broad-spectrum antibiotics in chronic wounds should be carefully restricted, as excessive and uncontrolled antibiotic use accelerates the development of MDR [38]. While the MRSA rate among *S. aureus* isolates was 12.5% in the current study, it was quite low at 4.9% in the study by Tabour et al. [39], whereas it was 14.7% in the study by Hasanpour et al. [40] and 17% in the study by Zhou et al. [41], which are levels similar to those in the current study. The low MRSA rate observed in the study by Tabour et al. may be attributed to the inclusion of patients from the emergency department, in addition to those from the chronic wound clinic.

The ESBL prevalence of 35.3% among *E. coli* isolates and 37.5% among *K. pneumoniae* isolates is clinically significant and consistent with the results of previous studies on wound and surgical site infections [42,43]. Since ESBL production is associated with plasmid-mediated resistance genes, this resistance mechanism may facilitate its spread in healthcare facilities and the community not only through clonal transmission but also via horizontal gene transfer [44,45]. The carbapenem resistance rate of 11.8% for *Pseudomonas aeruginosa* isolates is similar to the rates reported in previous study series [46,47]. In the current study, carbapenem resistance was detected in 66.7% of *A. baumannii* isolates. Although this rate is lower than that reported in a study from Türkiye, which found 100% carbapenem resistance [48], it is consistent with the literature showing that carbapenem resistance in *A. baumannii* is associated with limited treatment options, treatment failure, and adverse clinical outcomes [49,50].

In the current study, no significant differences were found in HbA1c, WBC, and CRP levels between groups with Gram-negative and Gram-positive microbial growth. Although this finding suggests that inflammatory markers may support the presence and severity of infection, it also indicates that they have limited value in predicting the Gram character of the isolate. Meta-analyses have emphasized that WBC and CRP levels are higher in infected diabetic foot ulcers; however, they cannot replace clinical judgment on their own [51,52].

Although there was a weak positive correlation between HbA1c, WBC, and CRP, the fact that no significant association was detected is consistent with the conflicting findings in the literature. While some studies have reported a significant association and prognostic value between CRP and HbA1c [53,54], other studies have shown that HbA1c does not independently predict recovery time and that inflammatory markers do not always correlate with clinical outcomes [55]. The moderate positive correlation observed between WBC and CRP in the current study supports the notion that both markers reflect systemic inflammatory burden in a similar manner. This finding is consistent with what would be expected in terms of the biological consistency of the inflammatory response [52].

### Limitations

The fact that the study was conducted at a single center limits the generalizability of the findings to other centers and different geographic regions. Due to the retrospective design, the data were limited to existing records; therefore, some important variables—such as comorbidities, prior antibiotic use, wound type, wound depth, and clinical outcomes—may not have been fully assessed for all cases. A further limitation is that although most patients were managed on an outpatient basis, a small subset were hospitalized, and in-hospital contamination or acquisition of resistant organisms cannot be excluded in this subgroup—particularly for Acinetobacter baumannii, a classic hospital-associated pathogen with a high rate of carbapenem resistance. Given the small size of this subgroup, a statistically meaningful comparison with community- and hospital-associated cultures was not possible. Another limitation is the absence of an independent control group; the study population consisted exclusively of patients who underwent wound culture sampling. As an alternative, isolates were categorized into Gram-negative and Gram-positive groups, but this classification is not equivalent to a true control and may carry a risk of selection bias and false-positive findings due to multiple comparisons. The observed associations should therefore be interpreted as hypothesis-generating rather than causal. Finally, data on prior or concomitant antibiotic exposure were not consistently available in the retrospective records. As chronic wound patients often receive repeated empiric antibiotics courses, this may have influenced the resistance patterns observed independently of the wounds’ intrinsic characteristics.

## 5. Conclusions

In conclusion, although the types of microorganisms varied depending on the wound type, the current study revealed a predominance of Gram-negative bacteria in chronic wound infections. While the overall MDR rate was limited and the MRSA rate was relatively low, the prevalence of ESBL-producing *E. coli* and *K. pneumoniae* isolates, along with the notably high prevalence of carbapenem-resistant *A. baumannii*, indicates that these are key resistance issues complicating infection management. Our findings support the need for local antibiotic susceptibility testing, regular resistance surveillance, and robust infection control measures in the selection of treatment.

## Figures and Tables

**Table 1:** Demographic and clinical characteristics of the patients.

Variable		Value	(%)
Age (years)	Median (min–max)	62.00 (18–96)	
Sex	Male	63	60.0
	Female	42	40.0
Wound culture growth	Single organism	70	66.7
	Polymicrobial growth	17	16.2
	No growth	18	17.1
Chronic disease *	Diabetes mellitus	68	64.8
	Hypertension	35	33.3
	Coronary artery disease	35	33.3
	Chronic renal failure	9	8.6
	Alzheimer’s disease/Dementia	7	6.7
	Other	40	38.1
Wound type	Diabetic foot ulcer	52	49.5
	Peripheral artery disease	30	28.6
	Pressure ulcer	15	14.3
	Peripheral venous insufficiency	5	4.8
	Other	3	2.9
Treatment type	Outpatient	97	92.4
	Inpatient	8	7.6
WBC (×10^3^/µL)	Median (min–max)	8.00 (4.20–31.60)	
HbA1c (%)	Median (min–max)	7.10 (4.80–16.60)	
CRP (mg/L)	Median (min–max)	23.30 (0.70–327.20)	

* Some patients had more than one chronic disease.

**Table 2:** Distribution of microorganisms isolated from wound cultures.

Microorganism	n	(%)
**Gram-negative microorganisms**	**73**	**70.2**
*Escherichia coli*	17	16.3
*Pseudomonas aeruginosa*	17	16.3
*Proteus* spp.	13	12.5
*Klebsiella* spp.	11	10.6
*Acinetobacter baumannii*	6	5.8
Other	6	5.8
*Citrobacter* spp.	3	2.9
**Gram-positive microorganisms**	**31**	**29.8**
*Staphylococcus aureus*	16	15.4
*Streptococcus* spp.	8	7.7
*Enterococcus* spp.	5	4.8
*Staphylococcus* spp.	2	1.9

**Table 3:** Antibiotic resistance of the isolated Gram-negative microorganisms.

Antibiotic (Overall n/%)	*E. coli* (n = 17)	*P. aeruginosa* (n = 17)	*A. baumannii* (n = 6)	*Proteus* spp. (n = 13)	*Citrobacter* spp. (n = 3)	*Klebsiella* spp. (n = 11)	Other Gram (−) (n = 6)
**Amikacin** *n = 7 (9.6%)*	–	–	5 (83.3%) *71.4%*	–	–	–	2 (33.3%) *28.6%*
**Amoxicillin–clavulanate** *n = 26 (35.6%)*	10 (58.8%) *38.5%*	–	–	8 (61.5%) *30.8%*	2 (66.7%) *7.7%*	3 (27.3%) *11.5%*	3 (50.0%) *11.5%*
**Ampicillin** *n = 37 (50.7%)*	12 (70.6%) *32.4%*	–	–	10 (76.9%) *27.0%*	2 (66.7%) *5.4%*	10 (90.9%) *27.0%*	3 (50.0%) *8.1%*
**Piperacillin–tazobactam** *n = 18 (24.7%)*	5 (29.4%) *27.8%*	2 (11.8%) *11.1%*	5 (83.3%) *27.8%*	1 (7.7%) *5.6%*	1 (33.3%) *5.6%*	4 (36.4%) *22.2%*	–
**Cefepime** *n = 9 (12.3%)*	4 (23.5%) *44.4%*	3 (17.6%) *33.3%*	–	1 (7.7%) *11.1%*	–	1 (9.1%) *11.1%*	–
**Cefoxitin** *n = 6 (8.2%)*	3 (17.6%) *50.0%*	–	–	–	1 (33.3%) *16.7%*	1 (9.1%) *16.7%*	1 (16.7%) *16.7%*
**Ceftazidime** *n = 14 (19.2%)*	6 (35.3%) *42.9%*	4 (23.5%) *28.6%*	–	1 (7.7%) *7.1%*	–	3 (27.3%) *21.4%*	–
**Ceftriaxone** *n = 14 (19.2%)*	7 (41.2%) *50.0%*	2 (11.8%) *14.3%*	–	2 (15.4%) *14.3%*	–	3 (27.3%) *21.4%*	–
**Cefuroxime** *n = 15 (20.5%)*	8 (47.1%) *53.3%*	–	–	3 (23.1%) *20.0%*	–	4 (36.4%) *26.7%*	–
**Ciprofloxacin** *n = 33 (45.2%)*	13 (76.5%) *39.4%*	5 (29.4%) *15.2%*	4 (66.7%) *12.1%*	5 (38.5%) *15.2%*	1 (33.3%) *3.0%*	3 (27.3%) *9.1%*	2 (33.3%) *6.1%*
**Trimethoprim–sulfamethoxazole** *n = 21 (28.8%)*	12 (70.6%) *57.1%*	–	2 (33.3%) *9.5%*	5 (38.5%) *23.8%*	–	1 (9.1%) *4.8%*	1 (16.7%) *4.8%*
**Imipenem** *n = 8 (11.0%)*	–	1 (5.9%) *12.5%*	4 (66.7%) *50.0%*	3 (23.1%) *37.5%*	–	–	–
**Meropenem** *n = 5 (6.8%)*	–	1 (5.9%) *20.0%*	3 (50.0%) *60.0%*	1 (7.7%) *20.0%*	–	–	–

Percentages in parentheses indicate the resistance rate of the given microorganism to that antibiotic; the percentage in the row below indicates the proportion of that microorganism among all isolates resistant to the corresponding antibiotic.

**Table 4:** Antibiotic resistance of the isolated Gram-positive microorganisms.

Antibiotic (Overall n/%)	*S. aureus* (n = 16)	*Enterococcus* spp. (n = 5)	*Staphylococcus* spp. (n = 2)	*Streptococcus* spp. (n = 8)
**Teicoplanin** *n = 1 (3.2%)*	–	1 (20.0%) *100.0%*	–	–
**Vancomycin** *n = 1 (3.2%)*	–	1 (20.0%) *100.0%*	–	–
**Levofloxacin** *n = 4 (12.9%)*	2 (12.5%) *50.0%*	2 (40%) *50.0%*	–	–
**Tigecycline** *n = 1 (3.2%)*	1 (6.3%) *100.0%*	–	–	–
**Benzylpenicillin** *n = 11 (35.5%)*	11 (68.8%) *100.0%*	–	–	–
**Clindamycin** *n = 11 (35.5%)*	4 (25.0%) *36.4%*	–	1 (50.0%) *9.1%*	6 (75.0%) *54.5%*
**Erythromycin** *n = 4 (12.9%)*	2 (12.5%) *50.0%*	–	–	1 (12.5%) *50.0%*
**Fusidic acid** *n = 6 (19.4%)*	4 (25.0%) *66.7%*	–	2 (100.0%) *33.3%*	–
**Mupirocin** *n = 2 (6.5%)*	2 (12.5%) *100.0%*	–	–	–
**Daptomycin** *n = 1 (3.2%)*	–	–	–	1 (12.5%) *100.0%*
**Oxacillin** *n = 2 (6.5%)*	1 (6.3%) *50.0%*	–	1 (50.0%) *50.0%*	–
**Tetracycline** *n = 13 (41.9%)*	6 (37.5%) *46.2%*	–	–	7 (87.5%) *53.8%*

Percentages in parentheses indicate the resistance rate of the given microorganism to that antibiotic; the percentage in the row below indicates the proportion of that microorganism among all isolates resistant to the corresponding antibiotic.

**Table 5:** Distribution of microbial isolates according to wound type.

Microorganism (Overall n/%)	Diabetic Foot Ulcer (n * = 54)	Peripheral Artery Disease (n * = 31)	Pressure Ulcer (n * = 14)	Peripheral Venous Insufficiency (n * = 3)	Other (n * = 2)
***E. coli*** *n = 17 (16.3%)*	11 (20.4%) *64.7%*	1 (3.2%) *5.9%*	5 (35.7%) *29.4%*	–	–
***P. aeruginosa*** *n = 17 (16.3%)*	5 (9.3%) *29.4%*	7 (22.6%) *41.2%*	4 (28.6%) *23.5%*	1 (33.3%) *5.9%*	–
***S. aureus*** *n = 16 (15.4%)*	12 (22.2%) *75.0%*	2 (6.5%) *12.5%*	–	1 (33.3%) *6.3%*	1 (50.0%) *6.3%*
***Proteus* spp.** *n = 13 (12.5%)*	9 (16.7%) *69.2%*	3 (9.7%) *23.1%*	1 (7.1%) *7.7%*	–	–
***Klebsiella* spp.** *n = 11 (10.6%)*	6 (11.1%) *54.5%*	4 (12.9%) *36.4%*	1 (7.1%) *9.1%*	–	–
***Streptococcus* spp.** *n = 8 (7.7%)*	6 (11.1%) *75.0%*	1 (3.2%) *12.5%*	–	1 (33.3%) *12.5%*	–
***A. baumannii*** *n = 6 (5.8%)*	1 (1.9%) *16.7%*	3 (9.7%) *50.0%*	1 (7.1%) *16.7%*	–	1 (50.0%) *16.7%*
***Other Gram (−)*** *n = 6 (5.8%)*	1 (1.9%) *16.7%*	4 (12.9%) *66.7%*	1 (1.9%) *16.7%*	–	–
***Enterococcus* spp.** *n = 5 (4.8%)*	2 (3.7%) *40.0%*	2 (6.5%) *40.0%*	1 (7.1%) *20.0%*	–	–
***Citrobacter* spp.** *n = 3 (2.9%)*	1 (1.9%) *33.3%*	2 (6.5%) *66.7%*	–	–	–
***Staphylococcus* spp.** *n = 2 (1.9%)*	–	2 (6.5%) *100.0%*	–	–	–

* Denotes the number of microorganisms isolated for the given wound type. Percentages in parentheses indicate the proportion of the given wound type in which that microorganism was isolated; the percentage in the row below indicates the proportion of that wound type among all isolates of the corresponding microorganism.

**Table 6:** Relationship between Gram-negative and Gram-positive microorganisms according to wound type.

Wound Type	Gram-Negative (n = 73)	Gram-Positive (n = 31)	Total (n = 104)	*p*-Value *
Diabetic Foot Ulcer	34 (46.6%) *63.0%*	20 (64.5%) *37.0%*	54 (51.9%) *100.0%*	0.055
Peripheral Artery Disease	24 (32.9%) *77.4%*	7 (22.6%) *22.6%*	31 (29.8%) *100.0%*	
Pressure Ulcer	13 (17.8%) *92.9%*	1 (3.2%) *7.1%*	14 (13.5%) *100.0%*	
Peripheral Venous Insufficiency	1 (1.4%) *33.3%*	2 (6.5%) *66.7%*	3 (2.9%) *100.0%*	
Other	1 (1.4%) *50.0%*	1 (3.2%) *50.0%*	2 (1.9%) *100.0%*	
Total	73 (100.0%)	31 (100.0%)	104 (100.0%)	–

* Data are presented as n (%). Fisher’s exact test was used because more than 50% of the expected cell counts in the Pearson chi-square test were below 5.

## Data Availability

The original contributions presented in this study are included in the article. Further inquiries can be directed to the corresponding author.
